# Privacy-preserving record linkage using Bloom filters

**DOI:** 10.1186/1472-6947-9-41

**Published:** 2009-08-25

**Authors:** Rainer Schnell, Tobias Bachteler, Jörg Reiher

**Affiliations:** 1 Methodology Research Unit, Department of Social Sciences, University of Duisburg-Essen, D-47057 Duisburg, Germany

## Abstract

**Background:**

Combining multiple databases with disjunctive or additional information on the same person is occurring increasingly throughout research. If unique identification numbers for these individuals are not available, probabilistic record linkage is used for the identification of matching record pairs. In many applications, identifiers have to be encrypted due to privacy concerns.

**Methods:**

A new protocol for privacy-preserving record linkage with encrypted identifiers allowing for errors in identifiers has been developed. The protocol is based on Bloom filters on *q*-grams of identifiers.

**Results:**

Tests on simulated and actual databases yield linkage results comparable to non-encrypted identifiers and superior to results from phonetic encodings.

**Conclusion:**

We proposed a protocol for privacy-preserving record linkage with encrypted identifiers allowing for errors in identifiers. Since the protocol can be easily enhanced and has a low computational burden, the protocol might be useful for many applications requiring privacy-preserving record linkage.

## Background

Combining multiple databases with disjunctive or additional information on the same person is occurring increasingly throughout medical research. More than 55% of approximately 3,400 entries in PubMed on "record linkage" have been published over the last 10 years. Since many studies in public health and epidemiology are based on surveys, it is surprising that there are now more entries in Pubmed on "record linkage" than on the combination of "survey" and "respondents". The availability of large medical databases and unique person identifier (ID) numbers has made widespread use of record linkage possible. But in many research applications not all databases contain a unique ID number. In such situations, probabilistic record linkage is most frequently applied for the identification of matching record pairs [1]. However, in many applications the identifiers have to be encrypted due to privacy concerns, which is problematic because linking encrypted identifiers can result in serious complications. Although there are some intriguing approaches proposed in the literature, these have a number of problems, for instance they involve very high computing demands or high rates of false positives or false negatives. Hence we developed a new procedure which addresses these problems.

### Introduction

Medical databases of people usually contain identifiers like surnames, given names, date of birth, and address information. Distribution of scientific files containing such information is legally restricted in most countries. The problem of finding records that represent the same individual in separate databases without revealing the identity of the individuals is called "privacy-preserving record linkage" [2], "blind data linkage" [3], or "private record linkage" [4]. Methods for privacy preserving record linkage can be subsumed under the general field of privacy preserving data integration [2] which also comprises a vast literature on privacy preserving database joining and querying [5-9]. However, this work either uses exact identifier comparisons or does not address the problem of generating micro data sets which are usable for general statistical purposes.

Initially, the obvious solution for privacy-preserving record linkage seems to be the encryption of the identifiers with a standard cryptographic procedure. An example is the Keyed-Hash Message Authentication Code (HMAC) introduced by [10]. The data holders apply a HMAC using a previously agreed secret key on the identifiers in their databases and send only the results of the HMAC (the hash values) to a third party [11]. Since the identifiers agree exactly if their corresponding hash values agree, the third party can link matching records without knowing the identifiers. Variants of this protocol using exact matching have been published [12,13].

Since these protocols require exact matching of identifiers, they do not tolerate any errors in these identifiers: Due to the design specifications of cryptographic functions, the slightest input variation results in many changes to the output (ideally, a change of one input bit should cause a change in half of the output bits). Applying probabilistic record linkage [1,14] improves the situation considerably since it does not require exact agreement in all (or even most) identifiers. Rather, agreements in strongly differentiating identifiers might balance disagreements in other identifiers. However, using string similarity functions within a probabilistic record linkage system will improve the linkage quality considerably. In addition, since records with variations of identifiers may have different characteristics to records with exact matching identifiers, restricting the linkage in this manner is not an option. Therefore, a method for approximate string matching in privacy-preserving record linkage is required. Originally, encoding identifiers phonetically before hashing them and using them with probabilistic record linkage procedures was suggested to achieve this [15,16]. In a seminal paper, Churches and Christen [11] recommended creating bigrams before hashing thereby allowing one to calculate bigram similarity scores between identifiers. The motivation on which these suggestions are based is to transform the identifiers in a manner that allows consideration of string similarities in a probabilistic record linkage procedure despite encrypting them. The aim of our paper is to describe a new method for the calculation of the similarity between two encrypted strings for use in probabilistic record linkage procedures.

### Related work

Several methods for approximate string matching in privacy-preserving record linkage have been proposed (for reviews see [12,17,18]). The protocols can be classified into protocols with or without a trusted third party.

#### Three-party protocols

Some protocols rely on exact matching of encrypted keys based on phonetically transformed identifiers by a third party. Such protocols are used for cancer registries [19,20] and information exchange between hospitals. In the proposal of [15,16] identifiers are transformed according to phonetic rules and subsequently encrypted with a one-way hash function. To prevent some cryptographic attacks on this protocol, the identifiers are combined with a common pad before hashing. The hash values are transferred to a third party who hashes them again using another pad. Then the third party performs exact matching on the resulting hash values. Despite exact matching, the linkage allows for some errors in identifiers, because hash values of phonetic encodings are matched. Providing database owners do not collude with the third party the protocol is secure. However, string comparison using phonetic encodings usually yields more false positive links than string similarity functions [21-23].

[11] suggested a protocol based on hashed values of sets of consecutive letters (*q*-grams, see below). For each string, the database holders *A *and *B *create for each record the power set of the *q*-grams of their identifiers. Each subset of the power set is hashed by an HMAC algorithm using a common secret key of the database owners. *A *and *B *form tuples containing the hash values, the number of *q*-grams in the hashed subset and the total number of *q*-grams and an encryption of the identifiers to a third party *C*. The number of tuples is much larger than the number of records. To calculate the string similarity between two strings *a *and *b*, *C *computes a similarity measure based on the information in the tuples. As [11] shows, *C *is able to determine a similarity measure of *a *and *b *by selecting the highest similarity coefficient of the tuples associated with *a *and *b*. To prevent frequency attacks, [11] propose to use an additional trusted party, thereby extending the number of parties involved to four. Furthermore, they recommend hiding the tuples among tuples created from dummy strings using Rivest's "chaffing and winnowing" technique [24]. Apart from an increase of computational and communication costs [25,26], the protocol is prone to frequency attacks on the hashes of the *q*-gram subsets with just one *q*-gram [17,18].

[27] used the value of a second identifier for padding every single character of a string before encryption. Subsequently, a third party is able to compare strings on the character level and to compute a string similarity. This elegant protocol requires a total flawless second identifier. However, a second identifier with few different values is open to a frequency attack.

In the protocol of [28] two data holders, holding lists of names, build an embedding space from random strings and embed their respective strings therein using the SparseMap method [29,30]. Then, each data holder sends the embedded strings to a third party which determines their similarity. To create the embedding space, data holder *A *generates *n *random strings and builds *z *reference sets from them. Next, *A *reduces the number of reference sets by the greedy resampling heuristic of SparseMap to the best *k *<* z *reference sets. These *k *reference sets are used to embed the names in a *k*-dimensional space. The coordinates for a given name are approximations of the distances between the name to the closest random string in each of the *k *reference sets in terms of the edit distance. As a result, for each name *A *receives a *k*-dimensional vector. After receiving the *k *reference sets from *A*, *B *embeds his names in the same way. Finally, both data holders send their vectors to a third party, *C*, who compares them using the standard

Euclidean distance between them. Using SparseMap allows the mapping of strings into the vector space avoiding prohibitive computational costs. This is accomplished by the reduction of dimensions using the greedy resampling method and by the distance approximations. However, the experiments in [28] indicate that the linkage quality is significantly affected by applying the greedy resampling heuristic.

Pang and Hansen [31] suggested a protocol based on a set of reference strings common to *A *and *B*. For a given identifier, both database holders compute the distances, *d*, between each identifier string and all reference strings in the set. If *d *is less than a threshold *δ*, the respective reference string is encrypted using a key previously agreed on by *A *and *B*. For each identifier string, the resulting set of encrypted reference strings along with their distances, *d*, and an ID number form a tuple. Both database holders send their tuples to a third party *C*. For every pair of ID numbers where the encrypted reference strings agree, *C *sums the distances, *d*, and finds the minimum of this sum. If this minimum lies below a second threshold *δ*_*sim*_, the two original identifier strings are classified as a match. The performance of the protocol depends crucially on the set of reference strings. Unless this is a superset of the original strings the performance is rather discouraging.

A different approach to solve the privacy-preserving record linkage problem for numerical keys is taken by [32]. They suggest using anonymized versions of the data sets for a first linkage step that is capable of classifying a large portion of record pairs correctly as matches or mismatches. Only those pairs which cannot be classified as matches or mismatches will be used in a costly secure multi-party protocol for computing similarities.

#### Two-party protocols

[33] suggested a protocol that allows two parties to compute the distance between two strings without exchanging them. Due to the large amount of necessary communication to compare two strings, such a protocol is unsuited for tasks with large lists of strings as required by privacy-preserving record linkage [28]. The protocol suggested by [34] uses a secure set intersection protocol described in [35]. However, this protocol requires extensive computations and is therefore also regarded as impractical for linking large databases [4,28].

The protocol of Yakout et al. [36] assumes that the data holders have already transformed their names into vectors as described by Scannapieco et al. [28] and is designed to compare them without resorting to a third party. In the first phase, the two data holders reduce the number of candidate string pairs by omitting pairs which are unlikely to be similar. In the second phase of the protocol, the standard

Euclidean distance between the remaining candidate vector pairs is computed using a secure scalar product protocol. Yakout et al. demonstrate that neither party must reveal their vectors in the computations. Although more parsimoneous, this protocol cannot outperform the protocol of Scannapieco et al. [28].

## Results

### Calculating string similarities using Bloom filters

The core problem of a privacy-preserving record linkage protocol is the calculation of the similarity of two encrypted strings. We suggest the use of Bloom filters for solving this problem. A Bloom filter is a data structure proposed by Bloom [37] for checking set membership efficiently [38]. Bloom filters can also be used to determine whether two sets approximately match [39].

#### Outline of the method

Suppose the similarity of two surnames should be computed. At first, both surnames are split into sets of consecutive letters (*q*-grams). Using 2-grams (usually called bigrams), the 2-gram similarity between the input strings _SMITH_ and _SMYTH_ (padded on both sides with blanks) can be computed with the Dice coefficient as , because each of these strings has 6 bigrams and the strings share 4 bigrams.

If we want to compute the similarity between those strings without revealing the bigrams, we must use an encryption. Our protocol for privacy-preserving record linkage uses a Bloom filter for this task. To accomplish this, we store the *q*-grams of each name in a separate bit array (a Bloom filter) using *k *multiple cryptographic mappings (hash functions) respectively. Then we compare the Bloom filters bit by bit and calculate a similarity coefficient.

**Figure 1:**
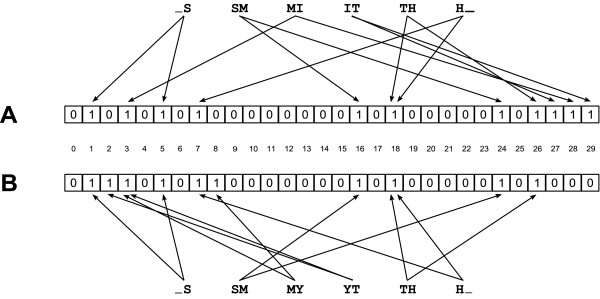
**Example of the use of two Bloom filters for the privacy-preserving computation of string similarities**.

Figure 1 illustrates the procedure for the two surnames SMITH and SMYTH using 2-grams, Bloom filters with a bit array of length 30, and two hash functions. The surnames are split into 2-grams and each of the resulting 2-grams is stored in the Bloom filters *A *and *B*. For example, the 2-gram _S (common to both names) yields the value 1 for the first hash function and the value 5 for the second hash function: The bits on positions 1 and 5 are set to 1 in both Bloom filters. In contrast, the 2-grams YT (hash values 2 and 3) and IT (hash values 27 and 29) occur in only one string and consequently different bit positions are set to 1. After mapping all bigrams to the Bloom filters, 8 identical bit positions are set to 1 in both Bloom filters. In total, 11 bits in *A *and 10 bits in *B *are set to 1. Using the Dice coefficient, the similarity of the two Bloom filters is . Therefore, the similarity between two strings can be approximated by using the Bloom filters alone.

Since cryptographic (one-way) hash functions are used, the initial input strings (names) can not be reconstructed given only the resulting Bloom filters. Therefore, record linkage by a third party or the research team is possible despite the privacy of the initial identifiers.

#### Implementation details

##### Bloom filters

A Bloom filter is a bit array of length *l *with all bits initially set to 0. Furthermore, *k *independent hash functions *h*_1_,..., *h*_*k *_are defined, each mapping on the domain between 0 and *l *- 1. In order to store the set *S *= {*x*_1_, *x*_2_,..., *x*_*n*_} in the Bloom filter, each element *x*_*i *_∈ *S *is hash coded using the *k *hash functions and all bits having indices *h*_*j *_(*x*_*i*_) for 1 ≤ *j *≤ *k *are set to 1. If a bit was set to 1 before, no change is made.

In general, set membership can be checked by hashing the candidate element *y *using the same *k *hash functions. If all bits having indices *h*_*i*_(*y*) in the Bloom filter are already set to 1, *y *is presumably a member of the set *S*. There is a probability *f *that the check indicates membership of *y *in *S *when in fact it is not. It is obvious that the probability of false positive cases depends on the bit array length *l*, the number of hash functions *k*, and the number of elements in *S *denoted by *n *(see [40]):(1)

On the other hand, if at least one of the bits is found to be 0, *y *is definitely not a member of the set *S*.

##### Hash functions

To store the *q*-grams in the Bloom filters, the double hashing scheme proposed by [41] was applied. They show that only two independent hash functions are necessary to implement a Bloom filter with *k *hash functions without any increase in the asymptotic false positive probability [41]. Therefore, *k *hash values are computed with the function(2)

where *i *ranges from 0 to *k *- 1 and *l *is the length of the bit array. For testing the algorithm, we used the well known cryptographic hash functions SHA1 (*h*_1_) and MD5 (*h*_2_) [42] in our implementation. In order to store surnames in a Bloom filter, decisions on the length of the bit arrays *l*, and the number of hash functions *k *must be made. The choice of these parameters is discussed below.

##### Similarity measure

If two surnames have many *q*-grams in common, their Bloom filters will have a large number of identical bit positions set to 1. Since the proportion of zeros in a Bloom filter for *n q*-grams is approximately [40]:(3)

a long Bloom filter will contain mostly zeros. To assess the similarity of Bloom filters, a coefficient insensitive to many matching zeros is desirable. Therefore the Dice-coefficient [43] was chosen. For comparing bit strings, the Dice-coefficient can be defined as(4)

where *h *is the number of bit positions set to 1 in both bit strings, *a *is the number of bit positions set to 1 in *A *and *b *the number of bit positions set to 1 in *B*.

## Testing

The performance of the new method was compared with the performance of the *q*-gram similarity between unencrypted surnames using simulated and actual databases.

### Comparison methods and criteria

As is the norm in the information retrieval literature, the criteria outlined by [44,45] were used to determine the recall and precision of the new methods. For a given level of similarity *φ*, a pair of records is considered as a match if the pair is actually a true pair, all other pairs are called non-matches [46]. Based on the common classification for true positive (*TP*), false positive (*FP*), false negative (*FN*) and true negative (*TN*) pairs, the comparison criteria are defined as(5)(6)

Plotting precision and recall for different similarity values *f *as a curve in a precision-recall-plot shows the performance of a string comparison method. A procedure with a better performance will have a curve in the upper right of the plot.

### Tests on simulated databases

In order to test the effect of different numbers (*k *= 5, 10, 25, 50) of hash functions on the performance of Bloom filters, similarities based on Bloom filters with a fixed filter length of 1,000 bits were compared with similarities based on unencrypted 3-grams (trigrams) of simulated data.

Additionally, we compared the proposed method to existing ones using a simulated database with a large number of records. We tested various string comparison methods within the same probabilistic record linkage procedure and compared the linkage quality of each setting. As string comparison methods we, used the Bloom filter method with a filter length of 1,000 bits and 30 hash functions, exact string comparisons and the Soundex phonetic encoding method [47].

#### Procedures and data sets

For the first simulation study, 1,000 surnames were sampled from the electronic German phone book. Lines containing just a single character were removed, umlauts and the German "β" converted and blanks, non alphabetic characters and most common surname components like "von" were deleted. A second list of names to be matched with the original surnames was generated in a copy of the file by changing exactly one character per name with probability *p *= .2 at a randomly chosen position in the name. Therefore two files with 1,000 surnames 20% of which differ at one position were used for computing string similarities. For the second simulation study, we created a large test database containing more realistic types of string errors. We used the test data generator implemented in Febrl [48] with its default settings since we regard them as quite realistic. First, we generated 500,000 artificial records containing identifiers such as given name, surname, title, address, sex, suburb, postcode, and age. Then we created a second database of the same size, resembling the first but containing 125,000 modified records. Amongst other error types, the test data generator allows one to insert and delete character values, to transpose two adjacent characters, to swap record fields and words, and to introduce common misspellings of the strings found in the records. We used given name, surname, address, street number, sex and suburb as comparison variables for matching. The same parameters for probabilistic record linkage were used when testing the different string comparison methods.

#### Results for simulated databases

**Figure 2:**
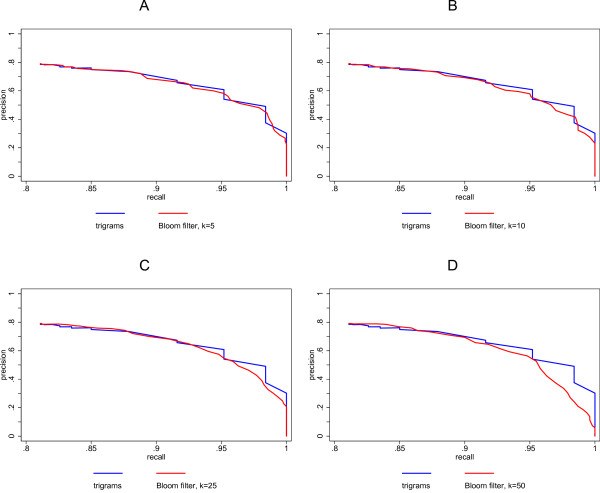
**Comparison of precision and recall for Bloom filters with unencrypted trigrams using simulated data**.

Figure 2 shows the results for the first simulation study. The precision versus recall curves in figure 2A are very similar. This is due to the fact that the probability of different trigrams being mapped to the same bit is very low (this probability is given by equation 1), since just five hash functions map the trigrams of a name onto 1,000 bits. When 10 hash functions are being used, the performance of the Bloom filter method is still very similar to the unencrypted trigrams (figure 2B). A small difference between the methods can be seen with 25 hash functions (figure 2C). However, even for 50 hash functions, the difference is not large (figure 2D).

To summarize: Inspection of the precision versus recall plots in figure 2 shows that if the number of hash functions is increased, the difference between the curve of the Bloom filter method and the curve of unencrypted trigrams also increases. However, at least up to 25 hash functions the Bloom filter method performs quite well compared with the unencrypted trigrams.

**Figure 3:**
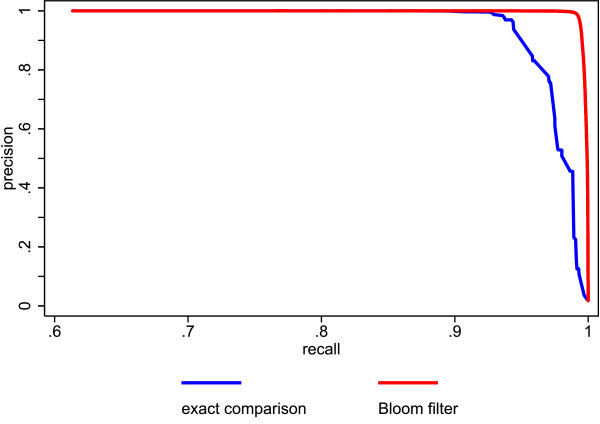
**Comparison of precision and recall for Bloom filters with exact string comparison using simulated data**.

**Figure 4:**
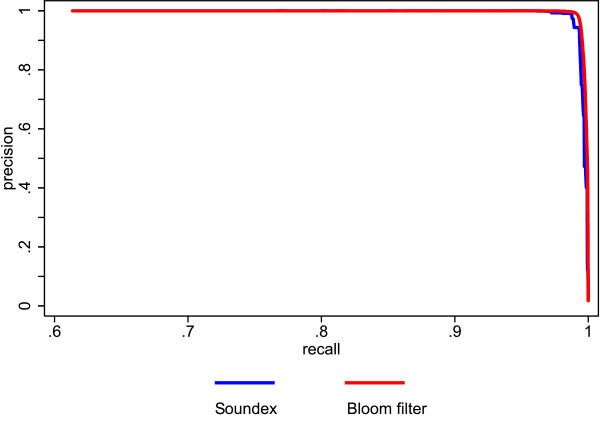
**Comparison of precision and recall for Bloom filters with Soundex using simulated data**.

The results of our second simulation study (this time using probabilistic record linkage) are shown in figures 3 and 4. Figure 3 shows the precision recall curves of the Bloom filter method and the exact string comparison. The Bloom filter method clearly outperforms the exact string comparison. Figure 4 depicts the precision recall curves of the Bloom filter method and Soundex. Again, the Bloom filter method is superior, although the difference is not as striking as before.

### Comparison of the Bloom filter method, unencrypted bigrams and a phonetic encoding using actual databases

In a second test, the performance of the proposed method was compared with the performances of unencrypted bigrams and a German phonetic encoding. This encoding (the so called "Kölner Phonetik") has been designed for matching German names and is widely used (for example, by the German cancer registries [20]).

#### Procedures and data sets

In the context of an evaluation of different probabilistic record linkage procedures for research purposes, we conducted a test of the proposed procedure on two German private administration databases. Each database contains identifiers of about 15,000 people. The task consisted of finding the intersection of the data sets. For this application we used bigrams with 15 hash functions on Bloom filters with 500 bits. The performances of the unencrypted bigrams, the phonetic encoding, and the Bloom filters was assessed by comparing the results of three complete record linkage runs with exactly the same parameters. The "Merge Toolbox" [49] was used for the record linkage.

#### Results for actual databases

**Figure 5:**
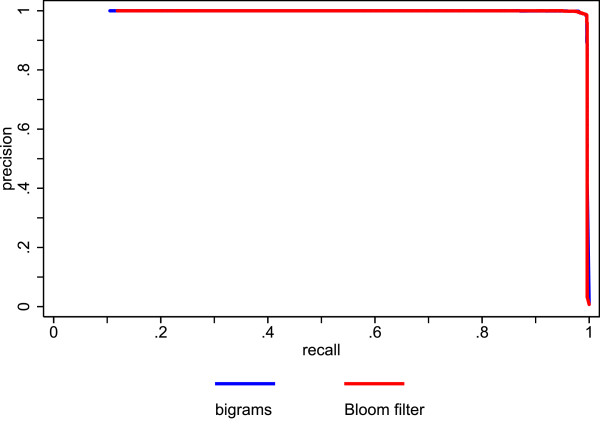
**Comparison of precision and recall for Bloom filters with unencrypted bigrams using actual data**.

**Figure 6:**
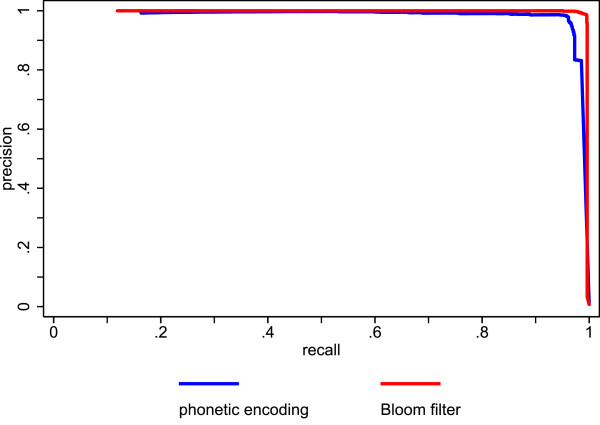
**Comparison of precision and recall for Bloom filters with a phonetic encoding using actual data**.

**Figure 7:**
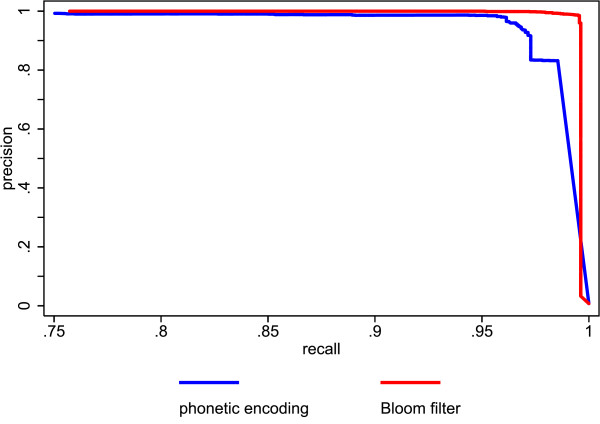
**Rescaled cutout of figure 6 highlighting recall levels above .75**.

Figure 5 shows the precision recall plots of the Bloom filter method and the unencrypted trigrams. The performance of the Bloom filter method is quite comparable to the performance of the unencrypted trigrams. Figure 6 shows the precision recall plots of the Bloom filter method and the German phonetic encoding. The Bloom filter method outperforms the phonetic encoding, especially at recall levels above .75 (figure 7 shows a cutout of figure 6 to highlight recall levels above .75). This is mainly due to the large number of false positives produced by the phonetic encoding.

## A Protocol for privacy-preserving record linkage

The previously described successful tests suggest the use of the method within a protocol for privacy-preserving record linkage. To add a layer of security, for an actual implementation of the Bloom filter method the hash functions SHA1 and MD5 should be replaced by a keyed hash message authentication code (HMAC) HMAC-MD5 and HMAC-SHA1 [50] with a secret key *K*. Based on this enhanced Bloom filter method, the implementation of a record linkage method is quite simple. Our protocol requires a third party, since each of the two database holders *A *and *B *could attempt a dictionary attack on the Bloom filters of the other party because they know the number of hash functions *k*, the secret key *K *and the length of the Bloom filters *l*.

Two database holders *A *and *B *with data sets *DB*_*a *_and *DB*_*b*_, a semi-trusted third party *C *and the recipient *D *of the merged data set participate in the protocol. *A *holds a list *S*_*a *_of *n*_*a *_strings, *B *holds a list *S*_*b *_of *n*_*b *_strings.

1. Data holders *A *and *B *agree on a bit array length *l*, on *k *hash functions, and a common secret key *K*.

2. For every string *i *in *S*_*a*_, *A *performs the following steps:

(a) *A *converts string *i *into the set of its *q*-grams.

(b) *A *stores the resulting *q*-gram set in a Bloom filter *bf*_*i *_of length *l *using the *k *keyed hash functions with the key *K*.

3. *A *stores the resulting *n*_*a *_Bloom filters and a randomly generated unique ID number *id*_*a *_in a list *BF*_*a*_.

4. *A *removes any identifier in *DB*_*a*_, replacing them by *id*_*a*_.

5. *A *sends *DB*_*a *_to D.

6. For every string *j *in *S*_*b*_, *B *performs the following steps:

(a) *B *converts string *j *into the set of its *q*-grams.

(b) *B *stores the resulting *q*-gram set in a Bloom filter *bf*_*j *_of length *l *using the *k *keyed hash functions with the key *K*.

7. *B *stores the resulting *n*_*b *_Bloom filters and a randomly generated unique ID number *id*_*b *_in a list *BF*_*b*_.

8. *B *removes any identifier in *DB*_*b*_, replacing them by *id*_*b*_.

9. *B *sends *DB*_*b *_to *D*.

10. *A *and *B *transfer the lists *BF*_*a *_and *BF*_*b *_to *C*.

11. *C *compares all possible pairs of Bloom filters in *BF*_*a *_× *BF*_*b *_counting the number of matching bits set to 1. The Dice similarity of the Bloom filters is used for finding the best matching pairs.

12. *C *sends the list of best matching pairs *BM *consisting of the tupels (*id*_*a*_, *id*_*b*_) to *D*.

13. *D *merges the files *DB*_*a *_and *DB*_*b *_using *BM*.

Our protocol is intended to match string attributes only. However, as outlined above, there are various protocols for matching attributes exactly in a privacy preserving manner. These protocols can be used for the comparison of numerical attributes in combination.

## Discussion

With respect to the data holders *A *and *B *the protocol is secure since neither holders have access to each others' Bloom filters. The third party *C *observes the Bloom filters of the data holders, but the encoding of the names is irreversible since the data holders use one-way hash functions to store the *q*-gram sets in the Bloom filters. Providing the data holders do not collude with the third party, a dictionary attack by *C *is impossible since the data holders use keyed one-way hash functions.

However, *C *could mount a frequency attack on the Bloom filters since the frequencies of the bit positions set to 1 will reproduce the frequencies of *q*-grams in the original strings. Padding the strings before splitting them into *q*-grams will worsen the situation since the *q*-grams containing pads will be more frequent. Obviously, the success of a frequency attack depends on the ratio of the number of hash functions used to the number of bits the Bloom filters are initialized with. Given the Bloom filter length, the more hash functions the data holders use, the more *q*-grams will share some bits set to 1 in the Bloom filter and the more difficult a frequency attack on the Bloom filters will be. Adding dummy strings and thereby additional Bloom filters using Rivest's "chaffing and winnowing" technique [24] in a manner that masks the original frequency distribution of bit positions set to 1 will provide additional security.

Bloom filters of short strings might be especially problematic since only a few bits are set to 1. Adding random *q*-grams or adding random bits to the Bloom filters could mitigate this problem.

Of course, the recipient *D *has a greater chance of re-identifying individuals from the merged data file as compared to the individual files *DB*_*a *_and *DB*_*b*_. However, this pertains to any kind of data linkage and is not an intrinsic problem of the proposed method. Any real world application of record linkage protocols has to guarantee factual anonymity of the resulting micro data sets. This can be achieved in many different ways. A technical option would be the use of micro data disclosure control algorithms [51].

Another problem might be if the recipient *D *transfers the merged data file to one of the data holders. The latter would then be able to re-identify individuals in the other holder's data base. However, this threat is common to any linked data file. If such a transfer cannot be prevented by legal means, the only remedy for this problem is a second trusted party *E*. *E *supersedes the recipient *D *in the protocol, merges the files *DB*_*a *_and *DB*_*b *_in his place, and pertubates the merged data file using disclosure control algorithms. Afterwards, *E *transfers the pertubated file to the recipient *D*.

## Conclusion

We proposed a protocol for privacy-preserving record linkage with encrypted identifiers allowing for errors in identifiers. The protocol is based on similarity computations of Bloom filters with HMACs on *q*-grams. This method has been tested successfully on simulated and actual databases. The linkage results are comparable to non-encrypted identifiers and superior to phonetic encodings. Since the protocol can be easily enhanced and has a low computational burden, the protocol might be useful for many applications requiring privacy-preserving record linkage.

## Competing interests

The authors declare that they have no competing interests.

## Authors' contributions

RS directed the SAFELINK-project on record linkage algorithms. All authors were involved in designing the algorithm. JR did the programming of the protocol, RS implemented independently a test version. RS and TB designed the tests for the protocol, TB tested the protocol on simulated and actual databases, RS on simulated data. TB drafted the manuscript, RS wrote the final version. All authors contributed to the protocol and approved the final version of the manuscript.

## Pre-publication history

The pre-publication history for this paper can be accessed here:

http://www.biomedcentral.com/1472-6947/9/41/prepub
